# Ribosomal Antibiotics: Contemporary Challenges

**DOI:** 10.3390/antibiotics5030024

**Published:** 2016-06-29

**Authors:** Tamar Auerbach-Nevo, David Baram, Anat Bashan, Matthew Belousoff, Elinor Breiner, Chen Davidovich, Giuseppe Cimicata, Zohar Eyal, Yehuda Halfon, Miri Krupkin, Donna Matzov, Markus Metz, Mruwat Rufayda, Moshe Peretz, Ophir Pick, Erez Pyetan, Haim Rozenberg, Moran Shalev-Benami, Itai Wekselman, Raz Zarivach, Ella Zimmerman, Nofar Assis, Joel Bloch, Hadar Israeli, Rinat Kalaora, Lisha Lim, Ofir Sade-Falk, Tal Shapira, Leena Taha-Salaime, Hua Tang, Ada Yonath

**Affiliations:** Department of Structural Biology, Weizmann Institute, Rehovot 76100, Israel; tauerba@its.jnj.com (T.A.-N.); david@smzyme.com (D.B.); anat.bashan@weizmann.ac.il (A.B.); matthew.belousoff@monash.edu (M.B.); elinor.breiner@weizmann.ac.il (E.B.); Chen.Davidovich@monash.edu (C.D.); giuseppe.cimicata@gmail.com (G.C.); zohar.baram@weizmann.ac.il (Z.E.); Yehuda.Halfon@weizmann.ac.il (Y.H.); Miri.Krupkin@weizmann.ac.il (M.K.); Matzov.donna@weizmann.ac.il (D.M.); markusmetz.at@gmail.com (M.M.); Rufayda.Mruwat@weizmann.ac.il (M.R.); moshe.peretz@weizmann.ac.il (M.P.); ophir.pick@gmail.com (O.P.); erezpy@gmail.com (E.P.); Haim.Rozenberg@weizmann.ac.il (H.R.); benami.moran@gmail.com (M.S.-B.); Itai.Wekselman@weizmann.ac.il (I.W.); zarivach@bgu.ac.il (R.Z.); Ella.Zimmerman@weizmann.ac.il (E.Z.); nofar.assis@gmail.com (N.A.); joel.bloch@mol.biol.ethz.ch (J.B.); hadarisr@bgu.ac.il (H.I.); rinat.kalaora@gmail.com (R.K.); lishaqjl@gmail.com (L.L.); ofir.sadefalk@gmail.com (O.S.-F.); Tal.Shapira@weizmann.ac.il (T.S.); Leena.ta.salaime@gmail.com (L.T.-S.); hua2han2003@yahoo.com (H.T.)

**Keywords:** multi-drug resistance, microbiome, species-specific antibiotics susceptibility, novel degradable antibiotics

## Abstract

Most ribosomal antibiotics obstruct distinct ribosomal functions. In selected cases, in addition to paralyzing vital ribosomal tasks, some ribosomal antibiotics are involved in cellular regulation. Owing to the global rapid increase in the appearance of multi-drug resistance in pathogenic bacterial strains, and to the extremely slow progress in developing new antibiotics worldwide, it seems that, in addition to the traditional attempts at improving current antibiotics and the intensive screening for additional natural compounds, this field should undergo substantial conceptual revision. Here, we highlight several contemporary issues, including challenging the common preference of broad-range antibiotics; the marginal attention to alterations in the microbiome population resulting from antibiotics usage, and the insufficient awareness of ecological and environmental aspects of antibiotics usage. We also highlight recent advances in the identification of species-specific structural motifs that may be exploited for the design and the creation of novel, environmental friendly, degradable, antibiotic types, with a better distinction between pathogens and useful bacterial species in the microbiome. Thus, these studies are leading towards the design of “pathogen-specific antibiotics,” in contrast to the current preference of broad range antibiotics, partially because it requires significant efforts in speeding up the discovery of the unique species motifs as well as the clinical pathogen identification.

## 1. Introduction

The severe increase in antibiotic resistance and cross-resistance among pathogenic bacterial strains presents a significant health threat. Hence, focusing on the specific properties of antibiotics targets in pathogenic bacteria, and on the molecular mechanisms acquiring resistance to them, are of prime importance. So far, the main efforts to combat antibiotic resistance are based on attempts at the production of new antibiotics by various approaches, such as mining underexplored microbial niches or designing new chemical probes for improving the antibiotic performance of known molecular scaffolds, and, to a lesser extent, the development of novel therapeutic agents (for reviews, see [1]). Presently, most clinically useful antibiotics are either natural products, originated by microorganisms, or semi-synthetic compounds based on natural molecular scaffolds that are produced by microorganisms to aid their struggle for resources. Interestingly, antibiotic resistance genes have existed in microorganisms long before these compounds were discovered by humans and exploited for therapeutic and nutritional uses. For example, the Yanomami’s gut bacteria have evolved a diverse array of antibiotic-resistance genes, even though this mountain tribe had never ingested antibiotics, nor animals raised in their presence [2].

**Figure 1:**
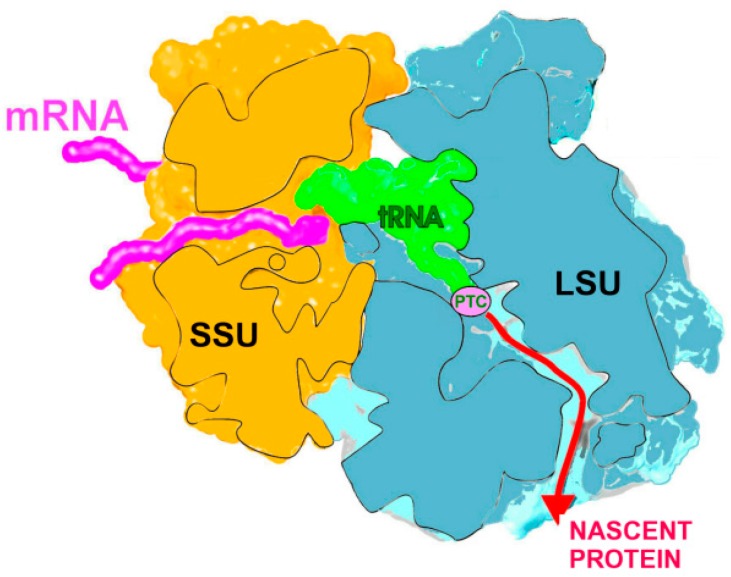
Overall structure of the bacterial ribosome showing the two subunits: the small (SSU) and the large (LSU), the mRNA, the PTC, and the nascent protein exit tunnel.

Protein biosynthesis is a key life process in all organisms; hence, it is targeted by many antibiotics. This process, namely, the translation of the genetic code, involves decoding the genetic information and the creation of nascent proteins. Ribosomes, the universal flexible and dynamic giant multi-protein-RNA assemblies, perform both tasks in all living cells, including pathogenic bacteria. They are built of two structurally independent riboprotein subunits that associate upon initiation of protein biosynthesis. In all organisms, the small subunit accommodates the mRNA and provides the site for the decoding of the genetic information, and the large subunit contains the site for peptide bond formation, called the peptidyl transferase center (PTC), and the tunnel along which the nascent proteins progress until they emerge from the ribosome (Figure 1). Owing to their key role in life, many clinically useful antibiotics paralyze them. Diverse mechanisms have been developed by microorganisms to acquire resistance to antibiotics, and it seems that, for most of them, the microorganisms generated specific antibiotic resistance genes. Here, we focus solely on those that are correlated to ribosomal antibiotics. These include post-transcription modification in the ribosomal RNA (rRNA) by specific enzymes (e.g., those that methylate or ethylate the C8 position of rRNA nucleotides) or by substitution and deletion/insertion mutations in ribosomal proteins (rProtein) that are located in proximity to the antibiotic binding pockets).

**Figure 2:**
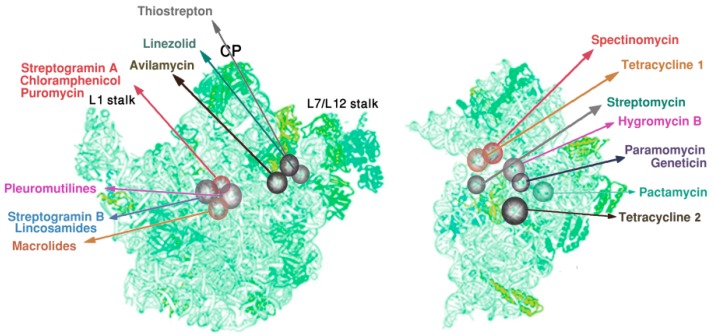
Main antibiotics binding sites shown on the skeletons of the large (**left**); and the small (**right**) ribosomal subunits.

Concurrent with the determination of the high-resolution structures of bacterial ribosomes, intensive efforts have been made to identify molecular modes of antimicrobial action, in order to reveal the principles of their selectivity, to shed light on their synergism, and to elucidate the mechanisms of acquiring resistance. Consequently, the target site and the mode of action of at least one member of each family of ribosomal antibiotics have been located and described in detail, showing that they inhibit protein biosynthesis by targeting functional regions in the ribosomes (Figure 2). Examples of targets in ribosomes, which were found or verified by X-ray crystallography, are the decoding region, the PTC, the nascent chain exit tunnel, an intersubunit bridge, and the tRNA accommodating corridor [3,4,5,6,7,8,9,10,11,12,13,14,15,16,17].

Species specificity of pathogens to antibiotics can be reached by several cellular pathways, such as efflux pumps and membrane permeability properties. Here, we focus only on the structural bases of ribosomal antibiotics binding and their modes of action. The crystallographic structural information provided valuable insights into the common mechanisms of antibiotic function, resistance, and selectivity that are shared by most of the clinically relevant bacteria, but did not show the minor structural differences between different pathogenic bacteria [18] that may be exploited for addressing species-specific significant differences in antibiotic susceptibility. In this regard, the structures of complexes of antibiotics with ribosomes from two pathogenic bacteria, *Escherichia coli* [7,8,15] and *Staphylococcus aureus* (*S. aureus*) [16], were shown to be useful for identification of unique structural motifs. Thus, comparisons of ribosome structures from pathogens with their non-pathogenic mates shed light on the properties of antibiotic action and resistance, and consequently paved new paths for dealing with the current acute resistance issues. In addition, a careful analysis of their modes of inhibition shed light on vital regulatory pathways and on ribosomal inherent flexibility.

Looking into the future, it is clear that there is an immediate necessity for novel anti-bacterial agents based on the specific atomic structures of targets in the ribosomes of pathogenic strains. Until recently, such data could only be obtained through lengthy and demanding crystallographic studies. However, the current resolution revolution by single-particle cryo-electron microscopy provides opportunities for relatively fast structure determination, smaller amounts, and the elimination of crystals. 

## 2. Main Findings

### 2.1. The Nascent Protein Exit Tunnel Seems to Be Invovled in Cellular Regulation

Erythromycin, the first ribosomal antibiotic drug that was used in clinical therapy, and the semi-synthetic compounds that are based on its chemical scaffold (including macrolides, ketolides, azalides, and streptogramin B), bind with a high affinity to a pocket, made solely of ribosomal RNA (rRNA) located at the rims of the nascent protein exit tunnel. Thus, the main macrolides mode of action is interfering with the progression of the nascent proteins [3,4,6,9,19,20,21,22,23,24] in a fashion that is still not fully characterized [23,24]. Resistance to macrolides is commonly achieved by mutation, e.g., A2058G (*E. coli* numbering is used throughout for rRNA nucleotides), or by modification, e.g., methylation, of the binding pocket’s nucleotides [25,26,27,28].

The protein exit tunnel is lined by rRNA and small regions of three ribosomal proteins (rProteins), among which uL4 and uL22 form the narrowest constriction of the tunnel. Each of them possesses a long β-hairpin loop, the tips of which are located in proximity, albeit chemically rather distal, to the macrolides binding pocket. Early biochemical, genetic, and structural studies revealed that explicit interactions of specific nascent protein sequence motifs with the tunnel walls may lead to translation arrest, thereby regulating the expression level of some genes [29,30,31,32,33,34,35,36,37,38,39,40]. The crystal structures of troleandomycin bound to the large ribosomal subunit [40] and of erythromycin resistant mutant with a minute deletion in protein uL22 [41] indicated a motion of the tip of the uL22 hairpin loop across the tunnel that could be correlated with the regulatory properties of the exit tunnel [40,41,42]. Furthermore, 2058A->G laboratory alteration mediates ketolide resistance in combination with a deletion in protein uL22. Hence, it is likely that erythromycin resistance mechanism can be involved with interfering cellular regulation, beyond a simple tunnel blockage.

Interplay between macrolides binding and translation arrest was also shown to control expression of macrolide resistance genes. Remarkably, it was found that macrolides arrest translation of a truncated regulatory peptide, even when the nascent chain was too short to encounter the antibiotic (the first three amino acids) [43]. Additionally, it was shown that stalling by specific nascent peptides, a cellular mechanism used for the regulation of expression of some bacterial and eukaryotic genes, is sensitive to additional cellular signals, some of which are connected to antibiotics [36,40]. These results correlate well with biochemical and structural findings that alterations of ribosomal components that do not belong to binding pockets may also cause resistance, mainly by exploiting the inherent flexibility of ribosomal components for reshaping the binding pockets or their environments [42,43,44].

### 2.2. Inherent Flexity of Antibiotics Binding Pockets and of Their Surroundings

One of the intriguing questions in antibiotic action relates to their selectivity, even when bound to almost fully conserved regions. An example is the PTC , which is highly conserved yet provides a binding site for several useful antibiotics, such as the pleuromutilin [45], lincosamides, phenicols [3] or small macrolides (e.g., a mono-sugar macrolide with 12 members macrolactone ring) [46]. Furthermore, the PTC undergoes indigenous post-translational modification that confers resistance to an array of protein synthesis inhibitors [47]. Additionally, the PTC possesses significant functional flexibility. Thus, crystal structures of complexes of the large ribosomal subunit with several pleuromutilins indicated that all pleuromutilins bind to the same PTC pocket, albeit by creating somewhat different interactions networks, and are all associated with a shift in the flexible PTC nucleotide U2585 and with an induced fit that tightens the pocket on the bound pleuromutilin antibiotics. The binding selectivity and some resistance mechanisms of this highly conserved region exploit non-conserved remote residues (e.g., those located in the second shell around the binding pocket) that may affect the conformation of nucleotides in the immediate vicinity of the binding pocket [5,48,49,50].

**Figure 3:**
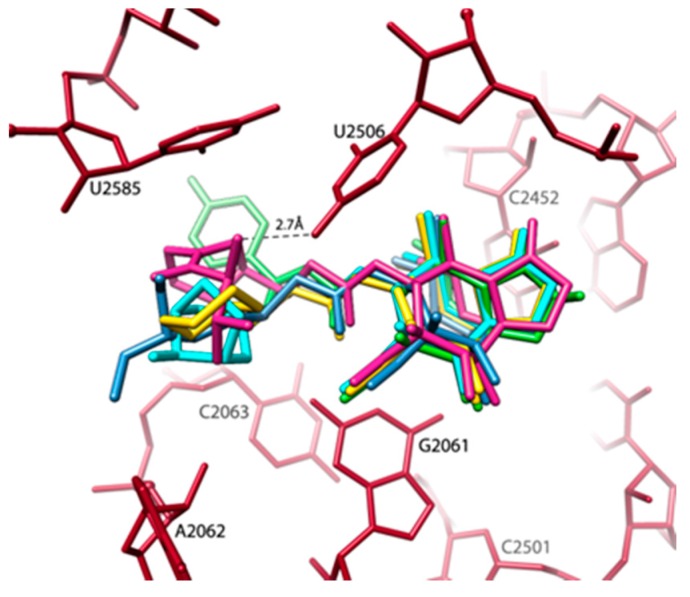
The pleuromutilins binding pocket in SA50S. Color code: SA rRNA-light brown, BC3205-violet, SB571579-green, Retapamulin-cyan, Tiamulin-slate, SB280080-yellow. The pleuromutilins antibiotics are superposed based on the locations of the rRNA of their binding pocket. The critical (additional) H = bond is shown.

Importantly, recent advances in pleuromutilin chemistry have yielded several new potential drugs; among them is BC-3205, a novel semisynthetic pleuromutilin derivative that was developed by Nabriva Therapeutics (Vienna, Austria) for intravenous or oral treatment of community-acquired bacterial pneumonia and skin infections. This compound is over 10 times more potent against *S. aureus* (SA) than most pleuromutilins and linezolid, and hence medically relevant. Interestingly, the crystal structure of the complex of the SA large ribosomal subunit (SA50S) indicated that this impressive improvement in potency is achieved by an addition of a single hydrogen bond between BC-3205 and the ribosome (Figure 3) [16].

### 2.3. Antibiotic Pairs and Synergism

**Figure 4:**
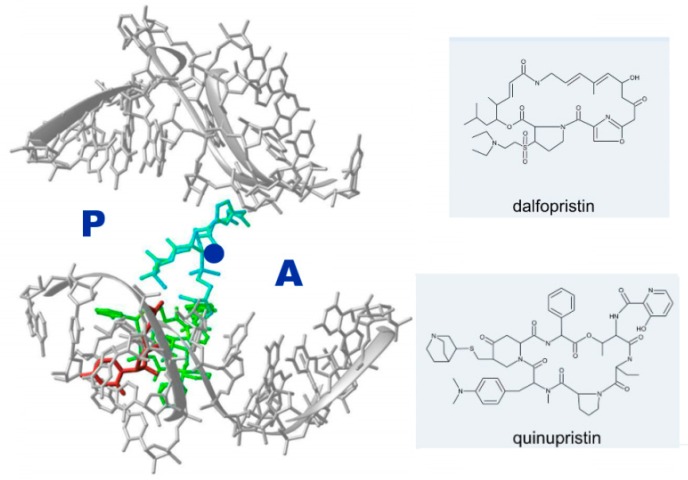
View into the PTC (rRNA backbone in grey), in which the approximate peptide bond formation position is marked by a blue circle, and the A- and P-sites tRNA 3′-ends are marked by A and P. Shown also are the locations of two components of Synercid™ (chemical formulas shown on the right side) within the PTC and in the tunnel’s entrance (dalfopristin in cyan and quinupristin in green). Note the swung location, out of the active site of nucleotide U2585 (in red).

A common resistance-acquiring mechanism is mutating the anchors between the antibiotic agent and its pocket. Hence, a potential approach to partially overcome (or reduce) resistance is to increase the number of anchors. This can be achieved by the use of pairs of antibiotics that bind to two different functional adjacent sites that can interfere synergistically with ribosomal function, although their synergy may occur independently of their individual effects on the translation [51]. Synercid™ is currently used as a synergetic pair against Gram-positive resistant strains, such as the methicillin-resistant *Staphylococcus aureus* (MSRA) and vancomycin-resistant *Enterococcus faecium* (VREF). This combined drug is made of a pair of semi-synthetic streptogramins, called dalfopristin (component A of Synercid™) and quinupristin (component B), which bind simultaneously to the PTC and to the macrolide binding pocket at the entrance to the exit tunnel, and exploit the inherent flexibility of these sites [52]. The synergistic effect of the streptogramins is driven by the streptogramin A (i.e., dalfopristin), which, upon binding to the 50S subunit, significantly increases the Kd of the streptogramin B (i.e., quinupristin) component. Importantly, the binding interactions network of streptogramin A is associated with a large shift of the essential flexible PTC nucleotide U2585, a motion that significantly alters the functional shape of the active site (Figure 4).

Although Synercid™ has been in use for a rather short period, resistance has already been reported. Hence, searches for additional synergetic pairs are being pursued. One of them is the lankamycin (LM)/lankacidin C (LC) pair, produced by a single organism, *Streptomyces rochei*, which uses dual genes harbored in a large plasmid. This dual production indicates a synergistic mode of action, which was found to display modest ability to inhibit cell growth as well as cell-free translation. In accordance with the biological results [52], the crystallographic structures of the large ribosomal subunit, in a complex with LM as well as with LM and LC together, indicate that their mechanisms for ribosomal inhibition are highly synergetic. It is based on LC binding to the PTC, thus preventing the proper placement of the aminoacyl end of the A-Site tRNA, whereas LM binds to the macrolides binding pocket and physically blocks the progression of the nascent peptides through the tunnel [38,53]. Both components of LM/LC and of Synercid™ bind to similar locations and significantly alter the functional shape of the PTC. Both indicate that flexible ribosomal nucleotides play a key role in drug binding. The stronger binding of LC aids the positioning of LM, thus allowing for synergistic inhibition of the ribosome function. Although the combination LM/LC is not as effective as Synercid™, their position within the ribosome provides clues to the development of more potent ribosomal interfering antibiotics.

### 2.4. Species Specificity and Susceptibility to Antibiotics

Overall, the binding and functional modes of action of antibiotic drug are common to all eubacteria, including most of those comprising the microbiome. Therefore, an unintentional outcome of the currently preferred broad-spectrum antibiotic treatments is modifying the delicate composition of the microbiome, which was found to be exceedingly influential in issues related to several significant diseases. Since species specificity in antibiotic susceptibility of various pathogenic bacteria targeting the large ribosomal subunit has been reported [54,55], it is suggested that responsiveness to the species-specific differences in drug action should minimize the uncontrolled, wild alterations of the microbiome.

**Figure 5:**
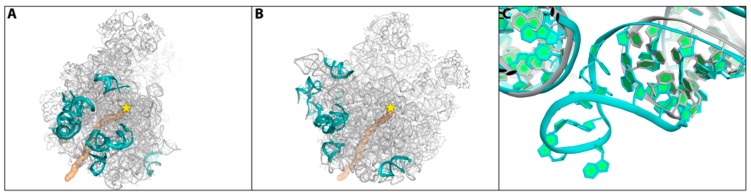
Left: The backbone of the large ribosomal subunit of SA50S is shown in gray from two views (face on (**A**) and a rotation of 90° (**B**)). The polypeptide exit tunnel is shown in gold and the PTC location is marked by a yellow star. The rRNA regions with fold variability compared with all other known structures on the SA50S surface are shown in cyan. Right (**C**): The stem loop of helix H63 in SA (cyan) and of *Deinococcus radiodurans* D50S (grey). Panel A & B are adapted from [16].

**Figure 6:**
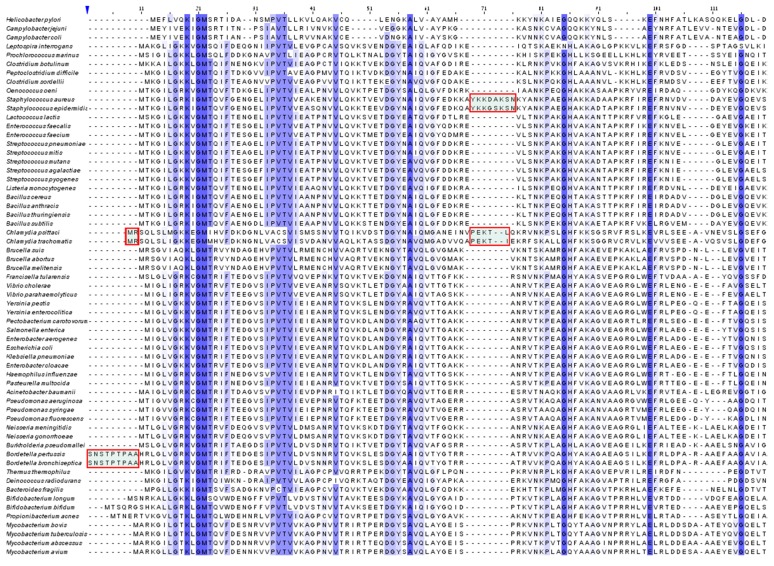
Multiple sequence alignment of protein uL3 from different bacterial species clearly showing its unique additional insertion (framed in red). The conserved sequences are highlighted in purple.

These expectations stimulated the structural studies on ribosomes from genuine pathogens and their careful comparisons to ribosomes from non-pathogenic species [16]. Consequently, the comparison between the structures of the large ribosomal subunit from non-pathogenic bacteria to those from *S. aureus* (SA) led to the identification of unique structural motifs that may be exploited for the design of advanced species-specific antibiotics [16]. Among others, considerable differences between the structures of ribosomes from non-pathogenic bacteria and that of *S. aureus* were detected in the stem loops of several rRNA helices (Figure 5). In parallel, differences were identified by structure and sequence alignments of ribosomal proteins, reflecting the ~50% sequence conservation between eubacterial rProteins. Among them is the medically important protein uL3 (Figure 6) [56,57], which contains a unique insertion segment of a few amino acids.

Consequently, it is suggested that species-specific structural motifs should be exploited as drug targets for better distinction between pathogens and the useful bacterial species in the microbiome, including the natural gut flora. This means aiming at the design of “pathogen-specific antibiotics” for each and every pathogen, in contrast to the current preference of broad-spectrum antibiotics. Furthermore, owing to the previous [16] and future identification of species-specific unique structural features, and to the recent indications of the importance of the content and variability of the microbiome, it is clear that the present goal in the field of ribosomal antibiotics should be to minimize antibiotic resistance alongside preserving the natural microbiome.

### 2.5. Ecological Aspects: Degradable Antibiotics

Many natural antibiotics are made of a scaffold, namely, an organic moiety core, to which one or more branches are covalently connected. Almost all of these scaffolds cannot be fully digested by humans or by animals (e.g., the macrolactone rings of erythromycin and of the various macrolides, ketolides and azalides; the central tricyclic mutilin ring of the pleuromutilins). Hence, their non-degradable metabolites, which are rather toxic, are reaching the environment, contaminating it, and thus increasing antibiotic resistance. 

Some of the newly identified potential sites, such as the extended and exposed rRNA helices, can, in principle, be exploited for the design of species-specific potent degradable antibiotics. For pursuing this idea, preliminary studies showed that compounds such as complementary DNA or PNA can interact with these sites and hamper in vitro translation. The ongoing research is based on previous experiments that showed that, oligonucleotides could be used as ribosomal inhibitors as well as tools for structural and functional studies. For example, short DNA oligonucleotides were used as “antisense DNA” to probe rRNA accessibility and for locating specific functional regions [58], before the three-dimensional structures of the ribosomes were determined. Such oligonucleotides can also potentially serve as the basis for future antibacterial drugs. Furthermore, in principle, these sites can also be probed by peptides, as performed recently by using the first 16 residues of mammalian Bac7 [59], or by molecules containing nucleic acids alongside amino acids, which can be optimized in terms of chemical properties and length. Thus, it is suggested that novel antibiotic drugs from degradable chemical components be designed, because their usage should hardly cause ecological or environmental contamination and consequently should reduce antibiotic resistance.

## 3. Conclusions

The studies reported here highlighted the interplay between internal flexibility, motion complementarity, cellular regulation, and antibiotic resistance. Furthermore, although so far based on a single, albeit important, example, focusing on selected acute issues in current antibiotics usage led to several unexpected and less obvious results, some of which are briefly mentioned below.

- Minimal inhibitory concentration (MIC) values of known antibiotics can be optimized. For example, the additional of a single hydrogen bond between an antibiotic and its pocket improves the MIC dramatically (Figure 3). Flexibility is a common property of antibiotics binding pockets. For example, in synergetic antibiotics, alterations of rRNA conformation proximal to the macrolide’s binding pockets can propagate towards the PTC. Additionally, it seems that there is an allosteric link between the tunnel and the catalytic center (PTC) of the ribosome.
- It is suggested that species-specific structural motifs should be exploited for the creation of novel antibiotics with a better distinction between pathogens and useful bacterial species in the microbiome. In fact, the next generation antibiotics should be degradable and species-specific. Thus, the aim of immediate research should be to minimize resistance to antibiotics while preserving the microbiome as well as reducing the contamination of the environment.
- The proposed design of “pathogen-specific antibiotics,” which is a revolution in the current concepts of antibiotics, is of immediate need. “Pathogen-specific antibiotics” means antibiotic drugs specific for each and every pathogen. This strategy requires the clinically fast identification of pathogens that is already being addressed [60].
- The practical application of “pathogen-specific antibiotics” requires the swift determination of the structures of antibiotics targets (e.g., ribosomes) of all or most pathogens. For this aim, the recent exciting development of single particle 3D cryo-electron microscopy should be more suitable than X-ray crystallography, since it can be performed by the use of relatively small amounts and does not require crystals.
